# 

**Published:** 1893-05

**Authors:** 


### LITERARY.

DIET FOR THE SICK.—By Miss E. Hibbard, Principal of Nurses' Training School, Grace Hospital, Detroit, and Mrs. Emma Drant, Matron of Michgan College of Medicine Hospital, Detroit; to which has been added Complete Diet Tables for various conditions. Detroit, Mich. The Illustrated Medical Journal Co., Publishers. Paper, 74 pp. Price, postpaid 25 cents, six for $1.00.

This little book is a worthy supplement to any cook book, as it deals only with the dishes suitable for the sick and convalescent. To this has been added the various authorized Diet Tables for use in various diseases. It also gives various nutritive enemas. The physician can use it to advantage in explaining his orders for suitable dishes for his patient, leaving the book with the nurse.

REVISED ENCYCLOPEDIA BRITANNICA; WORLD'S FAIR EDITION, Chicago Educational Publishing Co.

Every body knows the value of the above work, but all do not know that some 4000 biographies of distinguished people have been added to the American edition, of which Volume 1 is before us, which is a whole library in itself. The work will be published in weekly volumes after July 1, at $25.00 a year, till completed. Each volume will contain from 350 to 500 pages of matter, illustrated with colored maps, etc., sent to subscribers by mail, post paid.

No more valuable investment could be made for the money.

METHODS OF PRECISION IN THE INVESTIGATION OF DISORDERS OF DIGESTION, by J. H. Kellogg, M. D. Battle Creek, Mich., Publishing Company.

This is a pamphlet of 74 pp., which every student or practitioner of medicine will appreciate, and shows great care and precision in its tabulated statements and description of particular cases.

Dr. Kellogg is widely known to the profession as head of the famous Battle Creek Sanatarium.

CHOICE RECEIPTS.—By Miss Parloa, the well known founder of Schools of Instruction in Cooking. Specially prepared for Walter Baker & Co's. exhibit at the Columbian Exposition.

Every body knows of Baker's celebrated Breakfast Cocoa, but few know of the many ways of preparing chocolate for family use, explained in this interesting little pamphlet.

A REMARKABLE RESPIRATION RECORD IN INFANTILE PNEUMONIA. By William A. Edwards, M. D., San Diego, Cal., Fellow of the College of Physicians of Philadelphia, etc., etc.

SURGICAL DRESSINGS, ASEPTIC AND ANTISEPTIC. By Seward W. Williams, P H. C. ; F C. S.

UNIVERSITY OF PENNSYLVANIA.—The Catalogue and Announcement of this historical seat of learning, for 1892–3, is before us. Its inception goes back to 1749, inspired by a pamphlet by Doctor Franklin, and its first academic quarters were a building erected to accommodate the thronged congregations of the celebrated Whitfield. Its opening was in 1751, since which it continually grew in importance, until in 1757 its first college commencement was held, under a Provisional Charter obtained two years before. Its Provost, Mr. Smith, having incurred the ill will of the Pennsylvania legislature, was thrown into prison and actually received his classes there until liberated, that their studies might not be interrupted. Its university course embraces about every department of learning, with outside experiments in necromancy, not subversive of any useful purpose.

THE NEW YORK STANDARD.—We are in receipt of the April number of this interesting monthly, wherein some of our leading citizens have told the story of their " first watch." But Edison says he never carried a watch and never wanted to know what time it was, but we all know there was never but one Edison. All the rest of the world want watches. Our first watch would go only when it was carried. Something ailed its insides.

THE BANNER OF LIGHT.—Colby & Rich, publishers, Boston.—The Mid-April number of this able and excellent advocate of liberal ideas comes to us with twelve full pages of interesting and instructive matter. Admirable, friend Colby! you can never give us an overdose of good things, and such only we expect so long as you are in command.

